# Trypanosomiasis challenge estimation using the diminazene aceturate (Berenil) index in Zebu in Gabon

**DOI:** 10.1007/s11250-017-1239-2

**Published:** 2017-02-14

**Authors:** Brieuc G. A. Cossic, Brice Adjahoutonon, Pierre Gloaguen, Gui Lov Dibanganga, Gael Maganga, Pascal Leroy, Ewan T. MacLeod, Kim Picozzi

**Affiliations:** 1 Division of Infection and Pathway Medicine, Edinburgh Medical School: Biomedical Sciences, University of Edinburgh, Edinburgh, UK; 2 Société d’Investissement pour l’Agriculture Tropicale (SIAT-Gabon), Libreville, Gabon; 3 UMR MIA 518, INRA Versailles Grignon, Versailles, France; 4 Institut National Supérieur d’Agronomie et de Biotechnologies (INSAB), Franceville, Gabon; 5 Centre International de Recherches Médicales de Franceville, Franceville, Gabon; 6 Department of Animal Productions, Faculty of Veterinary Medicine, University of Liège, Liège, Belgium; 7 Tropical Veterinary Institute, Faculty of Veterinary Medicine, University of Liège, Liège, Belgium

**Keywords:** African animal trypanosomiasis, Berenil index, Diminazen-aceturate, Gabon, Tsetse, Zebu

## Abstract

A longitudinal study was conducted within a cattle ranch in Gabon to determine the diminazene aceturate (Berenil) index (DAI) in a group of Zebu, raised under low tsetse density; this measure providing an assessment of trypanosomiasis risk. The objective was to evaluate the trypanosomiasis pressure thus informing trypanosomiasis control methods and cattle management. Twenty female adult Zebu were monitored for 24 weeks during the dry season. Blood samples were collected on a weekly basis and subjected to parasitological and haematological analysis (*n* = 480), using the buffy-coat method and the packed cell volume value (PCV), respectively, infected animals were treated with a single intramuscular injection of diminazene aceturate (8 mg/kg). Twenty-nine single infectious events were recorded and a DAI of 1.45 was calculated. Two trypanosome species were identified: *Trypanosoma congolense* (96.2%) and *Trypanosoma vivax* (3.8%). The mean PCV value of the infected animals was lower (26.6) compared to non-infected animals (32.0). This study shows that DAI may be a useful tool to assess trypanosomiasis. However, this is a time-consuming method that may be improved by using randomly selected sentinel animals to adapt the chemoprophylactic schemes, hence decreasing the costs and the drug resistance risk.

## Introduction

African animal trypanosomiasis (AAT or nagana) is a disease endemic to much of sub-Saharan Africa, estimated to cost 35 million US dollars yearly to the owners of the 60 million cattle at risk of infection (Kristjanson et al. 1999; Swallow 2000). The disease is caused by a flagellated protozoan parasite belonging to the order Trypanosomatidae, genus *Trypanosoma*; namely *Trypanosoma congolense*, *Trypanosoma vivax* and to a lesser extent *Trypanosoma brucei* (Blood et al. 2007). Being a major obstacle to the development of animal breeding, AAT decreases the access to proteins of animal origin in countries where they are essential and where a large part of the population relies on livestock (de La Rocque et al. 2001).

The infection incubation period is 1 to 2 weeks; the first signs are often unnoticed and followed by a chronic phase with intermittent crises related to differential parasitemia that may lead to death by exhaustion after 2 to 3 months depending on the species of infectious agent (Bengaly et al. 2002). More generally, AAT will cause anaemia, cachexia and body condition loss. Intermittent fever attacks, oedema, abortion, emaciation, lymphadenopathy and a decreased fertility milk production and ability to work are also observed (OIE 2013; Murray et al. 1991). In the absence of a pathognomonic sign for AAT, diagnosis relies on laboratory methods to confirm the presence of the parasite. Although the polymerase chain reaction is currently the most sensitive mean of detection (Picozzi et al. 2002), the haematocrit centrifuge technique or Woo’s method (Woo 1970) and the buffy-coat technique or Murray’s method (Murray 1977) are well adapted to studies in-the-field corresponding to active infection, where confirmation of clinical cases and packed cell volume (PCV) are needed with minimum equipment and low costs.

Disease and vector control remain a considerable challenge with extensive areas to cover and great costs in money, time and manpower; therefore, an approach to assess the potential benefits from improved control has to be implemented (Shaw 2009). An estimated 17 million cattle are treated with trypanocides annually, assuming that animals are treated twice a year at a price of approximately one dollar per treatment, curative and preventive treatments would represent an estimated $35 million annual cost for the African livestock producer (Kristjanson et al. 1999). Within the context of this study, in an area with a low to medium prevalence according to the diagnostic method (Table 1) and using both curative and preventive trypanocidal drugs three times a year among trypanosensitive Zebu, represents an average cost of $4.2/year/Zebu (5.5% of a 10-year-old Zebu meat value). Moreover, trypanocidal drugs face a major difficulty with the appearance of drug resistance due to a widespread inadequate use of trypanocidal drugs (Mungube et al. 2012).

**Table 1:** Summary of African animal trypanosomiasis epidemiological studies conducted within the La Nyanga Ranch in Gabon

Year	Breed	Trypanosome prevalence	Diagnostic method	*Trypanosoma* species and prevalence
1987; Trail et al. (1990)	N’Dama	25%	BCM	*T. congolense* (65%), *T. vivax* (35%)
1988; Trail et al. (1990)	N’Dama	31%	BCM	*T. congolense* (74%), *T. vivax* (26%)
1989; Trail et al. (1990)	N’Dama	9%	BCM	*T. congolense* (64%), *T. vivax* (36%)
1985–1988; Ordner et al. (1988)	N’Dama	7.5; 10.1%	BCM	*T. congolense* (78 and 75%), *T. vivax* (22 and 23%)
1985–1988; Ordner et al. (1988)	Nguni	25.9%	BCM	*T. congolense* (68%), *T. vivax* (31%)
1985–1988; Ordner et al. (1988)	N’Dama × Nguni	16.5%	BCM	*T. congolense* (78%), *T. vivax* (22%)
1991; Leak et al. (1991)	N’Dama	5.4%	BCM	Both species detected in tsetse flies and cattle
2014; Maganga et al. (2016)	N’Dama	57.3%	ITS1 PCR	*T. congolense* (82.5%), *T. vivax* (17.5%)

(*BCM* buffy-coat method, *PCR* polymerase chain reaction)

To help in adapting anti-trypanosomal treatments to their local setting, Whiteside (1962) developed the diminazene aceturate (Berenil) index (DAI). This approach has been described by Leak (1999) as “a relatively simple way of measuring trypanosomiasis risk by measuring the frequency of infections in susceptible Zebu cattle (*Bos taurus indicus*) when each infection, as soon as it is detected, is treated with the trypanocidal drug, diminazene-aceturate (DA)”. It is a useful indicator of trypanosome risk that aids in defining treatment frequencies (Takken et al. 1988; Uilenberg 1998) and has the same accuracy as the collection of tsetse data (Claxton et al. 1991). DA is used because it has only short-term prophylactic activity, with an elimination half-life of 107.5 ± 8.50 h in calves (Kaur et al. 2000). Therefore, this study aims to calculate DAI in a small group of Zebu in order to adapt the treatment schemes at a local level under low tsetse density. The feasibility and efficiency of this method at a larger scale will be considered.

## Material and methods

The study area was located in the Ranch de La Nyanga in the southernmost province of Gabon. The ranch represents an area of 100,000 ha with a mean altitude of 150 m, a hilly landscape covered with herbaceous vegetation type and dotted with vegetation (*Brachiaria*, *Hyparrhenia*, *Panicum*, *Andropogon* and *Digitaria* species). Forest galleries are present along the gullies and rivers. Climate is equatorial with two dry seasons and two wet seasons. The average annual precipitation and temperature are of 2000 mm and 28 °C, respectively, with great yearly variations. The area was known for being a tsetse habitat in the 1990s with the presence of *Glossina tabaniformis*, *Glossina palpalis* and *Glossina nashi* at a low density (Leak et al. 1991).

From within the ranch herd, 20 6-year-old Zebu cows (with body weights 301 to 393 kg; average 352.5 kg (SD = 22.7)) were selected for inclusion in this research, to be initiated after the weaning of their last calf. Vaccination (pasteurellosis and contagious bovine pleuropneumonia) and deworming (ivermectin) were performed. Every 2 weeks, animals were dipped into a flumethrin bath to kill and repel ticks and tsetse flies; animals were weighed monthly, using weighing bars (Avery-Weigh Tronix Chute Weigh 1.75; 640 XL indicator). The programme lasted for 24 weeks between April 18 and October 3, 2014; on day one of the protocol, each animal received 8 mg/kg of DA, a dose at the top end of the recommended regimen (VERIBEN®, Ceva Africa).

Three millilitres of blood were collected from the coccygeal vein on a weekly basis into EDTA-treated blood collection tubes. Samples were kept in a cool box and transported to the ranch’s laboratory, stored in a refrigerator at 4 °C and processed the day after according to the recommendations of Woo (1970) and Murray (1977). Positive results were considered when at least one trypanosome was observed; when possible, the *Trypanosoma* species was determined. Negative results were considered when no parasite was observed. When infected, based on the results of the previous week, animals were treated with an intramuscular injection of DA (8 mg/kg). Once infected, an animal was sampled twice, the day of the first diagnosis and a week later, the day of the treatment. Therefore, a false negative was considered when an animal was positive for trypanosomiasis during the standard screening protocols (the first week), but negative upon follow-up 1 week later, with blood having been collected before trypanocidal treatment was given.

The DAI is calculated as follows: the number of infections recorded over the 6 months (Whiteside 1962), following the baseline administration of trypanocide, divided by the number of animals. Statistical analysis was conducted using the software “R” version 3.1.0.

## Results

A study was conducted over a 24-week period in a cattle ranch in Gabon to estimate the DAI and the *Trypanosoma* spp. prevalence among 20 Zebu; at baseline, 13 animals were infected, and following clearance of infection after the initial treatment with trypanocide a further, 29 instances of trypanosomiasis were recorded over the 6-month study period.

Eighteen Zebu out of 20 (90%) were positive for trypanosomiasis at least once during the observation period, with a total of 29 different infectious events and a DAI of 1.45 (29/20). Two species of *Trypanosoma* were observed, with 96.6 and 3.4% for *T. congolense* and *T. vivax*, respectively. The mean interval between two infections for the same animal was of 6.1 weeks, with a minimum period of 3 weeks following treatment being reported. Eleven multiple re-infections were observed after the baseline treatment, with six animals being infected twice, one being infected three times and one animal infected four times. Twelve false negative were identified, representing 21% of the 57 analyses performed upon animals identified as infected according to direct visualisation of trypanosomal parasites by microscopy.

**Fig. 1:**
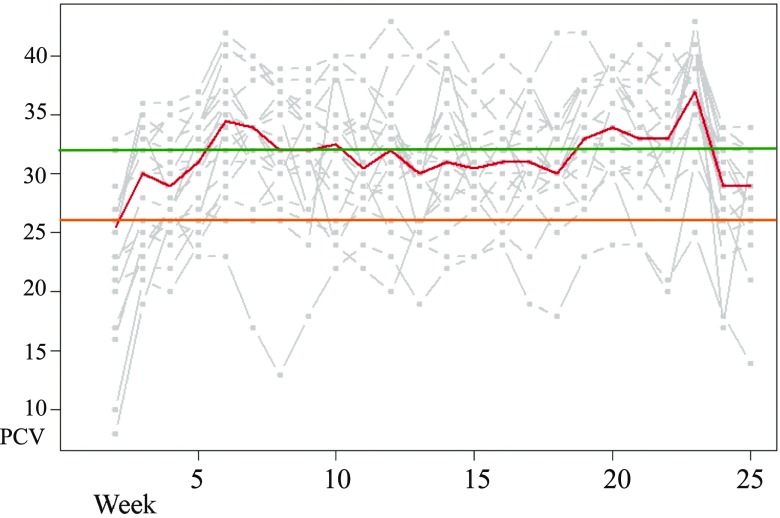
PCV values. The median for the herd is represented by the *central line*. The *boundaries* reflecting the mean PCV value for non-infected animals are represented by the *top line* (showing the constant value of 32) and the mean PCV value at the moment of the infection is on the *bottom line* (showing the constant value of 25.6)

PCV value results are represented on Fig. 1. On day one, the average PCV was 23.5 (SD = 6.6; min = 8; max = 33). Following mass treatment of the study cohort, the PCV had risen to 28.6 (SD = 5.1; min = 19; max = 36) 1 week later. At the end of the experiment, it was 27.8 (SD = 4.5; min = 14; max = 34). The PCV values for non-infected animals had a mean at 32.1 (SD = 4.8; min = 17; max = 43), whereas PCV value when infected is of 25.8 (SD = 5.9; min = 8; max = 36). A Student’s *t* test was performed and rejected the null hypothesis of equality of PCVs between groups (*P* value <0.001). On day one, the average weight was 352.5 kg (SD = 22.7; min = 301; max = 393). At the end, it was 373.3 kg (SD = 14.65; min = 345; max = 400). Interestingly, the animal with four infections also had the lowest weight at the end of the study period.

## Discussion

As demonstrated in this study, under low tsetse density, the weekly monitoring of cattle using microscopy was effective in detecting and controlling the occurrence of trypanosome infection within this population. However, this level of animal health management has a cost implication in terms of money, manpower and time; as well as the need for local diagnostic facilities.

The DAI can be used to establish the challenge of infection within a given area and represents the average number of infections each animal is likely to contract over a period. Most of the time, DAI index is determined over an annual period, which is considered as a minimum to have a representative result. According to Uilenberg (1998), over a year, a DAI of 3 for Zebu is relatively low and requires only curative treatment of infected animals detected on a monthly or two-monthly basis. A DAI of 4 to 6 indicates a medium to high challenge and requires monthly curative treatments or a prophylactic treatment. A DAI higher than 6 indicates a high to very high challenge and requires prophylactic treatment and the use of sensitive breed should be review.

Here, we report a DAI of 1.45 infections/cow/6 months, suggesting a low challenge of infection; our study occurred during the dry-season, when trypanosomiasis challenge is lower than during the rainy season and is considered in the presence of a fortnightly application of insect repellent. A previous study in Gabon, which focused upon Nguni cows (a cross-breed between *B. taurus indicus* and *B. taurus*) demonstrated a similar level of 3.2 infections/cow/year over a longer timespan (Ordner et al. 1988). Herein, the first re-infections occurred on average 6 weeks after the DA treatment, the minimum period between re-infection was 3 weeks. These observations highlight the absence of drug resistance and therefore the validity of the DAI within this setting and suggest that on average the elimination half-life of this treatment may be much longer than suggested by Kaur et al. (2000), being more in keeping with values of 188 and 222 h suggested by Kellner et al. (1985) and Gummow et al. (1994), respectively. It is noted that given the DAI recorded, the recommendation that infections can be managed by monthly screening would have resulted in a small number of infected animals being present within the herd (Uilenberg 1998).

The DAI is often underestimated because of the lack of sensitivity of the laboratory tests (Uilenberg 1998); the amount of false-negative results in this case (21%) confirms the lack of sensitivity of the microscopy to detect low parasitemia. According to Murray (1977) analyses should be performed soon after collection and at least within 4 to 6 h to limit the decrease in the number of detectable parasites, especially with *T. congolense*; it is even more important if the sample is collected between two peaks of parasitemia (OIE 2013). When conditions are good, a detection of parasites of almost 100% can be achieved following the Woo method when at least 700 trypanosomes/ml of blood are present. This decreases to 80–46% of detection between 700 and 60 parasites/ml and almost 0% below 60 tryps/ml for *T. vivax* (Desquesnes 2004). However, during the current study analyses have been performed 24 h after the sampling due to logistical restrictions. *T. congolense* is the most represented of the species (96.6%) identified, followed by *T. vivax* (3.4%). This is consistent with the species and proportions observed previously (Table 1); this species is deemed more pathogenic for cattle (Trail et al. 1990).

When laboratory facilities are limited, the Woo (1970) and Murray (1977) methods present the advantage of easy performance in the field with minimal training, few materials required and cheap consumables. The ongoing development technologies dedicated to a use in the field, such as Foldscope (Cybulski et al. 2014) and hand-powered centrifuges such as Paperfuge (Bhamla et al. 2017) aim to encourage quick examination of the samples in remote areas. However, the sensitivity may remain low and the prevalence underestimated; PCR in that matter would provide a great sensitivity and specificity (Maganga et al 2016; Gall et al. 2004) and great improvement are being made to have more affordable diagnosis, directly in the field (Marx 2015).

The mean PCV of 32.1 (SD = 4.8), for non-infected animals is equivalent to 34.9 described in the literature for adult Zebu (Merlin 1986). As expected, with a mean value of 25.8 (SD = 5.9), PCV values are lower for infected animals than for non-infected animals. On the first treatment day, after a 4-month period of unmonitored challenge, the average PCV was initially of 23.5 (SD = 6.6). The value rose to 28.6 (SD = 5.1) after only 1 week, which indicates a high efficiency of the DA and a high ability of the animals to recover, as described by Trail et al. (1990). Anaemia can be caused by AAT and is therefore an important indicator with 94% specificity and 89% sensitivity when a cut-off value of 26 is observed if combined to parasitological diagnosis (Marcotty et al. 2008). However, with only two non-infected animals, this is unlikely to be significant in this case.

Over the entire protocol, only two Zebu out of 20 remained clear of infections over the study period, compared to one animal within the same cohort that was infected four times. This highlights the possibilities for individual selection for trypanotolerance based on the DAI, along with other criteria (Traore 1989). For example, the increase in PCV and weight from the beginning to the end of the programme could be explained by better veterinary support throughout the programme with animals receiving prompt treatment to ailments. The study period also aligned with recovery after lactation, following weaning of the animals last calf prior to this commencement of this research.

Dia and Desquesnes (2007), in a *Rational Use of Drugs Guidance* manual described the management of trypanosomiasis in different situations; if the risk is low over the whole year, a targeted curative treatment for infected animals only is recommended. This recommendation would appear to be appropriate to the setting described above. The risk of infection, the seasonality of AAT and the presence of drug resistance help to define the most appropriate trypanocide strategy. Monitoring efficacy of a control programme using DAI determination is demonstrated to be a viable method of trypanosomiasis risk assessment within a localised setting. However, weekly monitoring of cattle is a slow and time-consuming operation with high costs in terms of manpower. At a larger scale, the use of 5–10 sentinel animals, with monthly review in each area, could be considered depending on the size of the herd.
